# Induction of ethylene inhibits development of soybean sudden death syndrome by inducing defense-related genes and reducing *Fusarium virguliforme* growth

**DOI:** 10.1371/journal.pone.0215653

**Published:** 2019-05-22

**Authors:** Noor A. Abdelsamad, Gustavo C. MacIntosh, Leonor F. S. Leandro

**Affiliations:** 1 San Joaquin Valley Agricultural Sciences Center, USDA-ARS, Parlier, CA, United States of America; 2 Roy J. Carver Department of Biochemistry, Biophysics and Molecular Biology, Iowa State University, Ames, IA, United States of America; 3 Department of Plant Pathology and Microbiology, Iowa State University, Ames, IA, United States of America; National Institute of Technology Rourkela, INDIA

## Abstract

Ethylene is a gaseous hormone that regulates plant responses to biotic and abiotic stresses. To investigate the importance of ethylene in soybean resistance to *Fusarium virguliforme* (*Fv*), the causal agent of sudden death syndrome (SDS), soybean cultivars Williams 82 (SDS-susceptible) and MN1606 (SDS-resistant) were treated 24 h before and 24h after *Fv* inoculation with either ethephon (ethylene inducer), cobalt chloride (ethylene biosynthesis inhibitor), or 1-MCP (ethylene perception inhibitor). Inoculated plants were grown for 21 days at 24°C in the greenhouse and then evaluated for SDS severity and expression of soybean defense genes. In both cultivars, plants treated with ethephon showed lower SDS foliar severity compared to the other treatments, whereas those treated with cobalt chloride or 1-MCP showed the same or higher SDS foliar severity compared to the water-treated control. Ethephon application resulted in activation of genes involved in ethylene biosynthesis, such as ethylene synthase (*ACS*) and ethylene oxidase (*ACO*), and genes involved in soybean defense response, such as pathogenesis-related protein (*PR*), basic peroxidase (*IPER*), chalcone synthase (*CHS*), and defense-associated transcription factors. Cobalt chloride and 1-MCP treatments had little or no effect on the expression of these genes. In addition, ethephon had a direct inhibitory effect on *in-vitro* growth of *Fv* on PDA media. Our results suggest that ethephon application inhibits SDS development directly by slowing *Fv* growth and/or by inducing soybean ethylene signaling and the expression of defense related genes.

## Introduction

Sudden death syndrome (SDS), caused by the soilborne fungus *Fusarium virguiforme* (*Fv*) [1] is one of the most damaging diseases to soybean production in North and South America. In the last two decades, SDS was ranked among the top ten most damaging soybean diseases in the United States, with average yield losses ranging from 0.3 to 2 million metric tons per year [2, 3]. As a soilborne pathogen, *Fv* infect roots at early soybean growth stages, causing root rot and reduction in root biomass. The fungus then releases phytotoxins that cause foliar interveinal chlorosis and necrosis and premature defoliation; these foliar symptoms usually appear during reproductive growth stages [4, 5]. Cool (15°C), wet soil early in the growing season, followed by intermediate temperatures (22–24°C) during soybean reproductive development, are favorable environmental conditions for SDS symptom development [6].

Host resistance is the most effective management practice against SDS. However, resistance to SDS is quantitative, i.e. is controlled by multiple genes, which adds complexity to plant breeding strategies aiming to accumulate numerous QTL into a single cultivar [7]. Other management strategies such as crop rotation, tillage, and delayed planting date are often inconsistent and have limitations [8].

Treatment of plants with synthetic chemical elicitors, such as hormones or their analogs, can induce resistance against a broad spectrum of plant pathogens, a phenomenon known as systemic resistance [9–11]. Induction of systemic resistance is controlled by plant hormones, such as salicylic acid (SA), jasmonic acid, and ethylene (ET) [10]. In general, SA is known to play an important role in activation of plant defense mechanisms against infection by biotrophic or hemibiotrophic pathogens, and is required for induction of systemic acquired resistance. In contrast, JA and ET play a crucial role in resistance against necrotrophic pathogens, and are required for induced systemic resistance [10, 12].

Ethylene is a gaseous hormone involved in multiple plant growth and developmental processes, as well as response to biotic and abiotic stresses [13]. Several studies showed that ethylene has a role in the development of disease resistance, as it induces the expression of phytoalexins and pathogenesis related (PR)-proteins [12, 14]. However, ethylene signaling pathways may act as a positive or negative regulators of disease resistance, depending on pathogen life style and plant species [15, 16]. For example, exogenous application of ethylene or ethephon (ethylene releasing substance) induces resistance against different pathogens, such as *Macrophomina phaseolina* in *Medicago truncatula* [17], *Magnaporthe oryzae* in rice [18], *Phytophthora capsici* in habanero pepper [13], and *Botrytis cinerea* in grapevine [15]. Plant mutants impaired in ethylene perception have also shown enhanced disease susceptibility, as reported for ethylene-insensitive tobacco plants inoculated with non-pathogenic soilborne fungi [16], and in ethylene insensitive soybean mutants infected with *Sclerotinia sclerotiorum*, *Septoria glycines* and *Rhizoctonia solani* [19, 20]. In contrast, other studies showed that ethylene may act as a virulence factor and play a role in disease development [21, 22]. For instance, soybean ethylene insensitive mutants developed less severe symptoms in response *Pseudomonas syringae* pv *glycinea*, and *Phytophthora sojae* [20].

Transcriptome analyses indicate that genes involves in ethylene biosynthesis are induced in response to *Fv* infection in soybean [23, 24]. However, it is not clear if this ethylene accumulation affected SDS resistance positively or negatively. In this study, we investigate the role of ethylene in the soybean-*Fv* interaction by manipulating ethylene accumulation and responses by the application of ethylene inducing and ethylene suppressing chemicals.

## Materials and methods

### Plant material

Two soybean [*Glycine max* (L.) Merrill] genotypes, Williams 82 (moderately susceptible to SDS) and MN1606 (resistant to SDS), were used in all experiments. Four seeds were sown 1 cm below the soil surface in 240 ml Styrofoam cups, then thinned to one seedling per cup after germination. The plants were incubated in a greenhouse bench at 24°C, with a 16-h photoperiod, watered as needed, and fertilized once a week.

### Pathogen culture

*Fv* isolate NE-305 was used as the inoculum source in all experiments. A single-spore *Fv* isolate was collected from an infected plant in Nevada, IA in 2006, and maintained in potato dextrose agar (PDA) media for long-term storage. For inoculum preparation, 21-day-old cultures on PDA were flooded with 20 ml of sterile distilled water (SDW), the conidia were dislodged with a rubber policeman, and the suspension was filtered through a double layer of sterile cheesecloth. The spore concentration was then adjusted to 10^6^ conidia/ml using SDW. The conidial suspension was used to infest a sand and cornmeal mixture, following the procedure described by Munkvold and O’Mara [25]. A mixture of 1900 ml of sand, 380 ml of cornmeal and 110 ml of SDW was autoclaved in 20 cm X 30 cm bags (Fisher scientific, Pittsburg, PA) for 1 hour at 121°C, on two consecutive days. Each bag was then amended with either 2 ml of the 10^6^-conidia/ml suspension, or with 2ml of SDW for the controls. The bags were incubated in the dark at room temperature for 6 days with daily mixing by hand, to keep a uniform distribution of *Fv*.

### Effect of chemical treatment on soybean SDS in the greenhouse

#### Experimental design

A greenhouse experiment was conducted to test the effect of ethephon, cobalt chloride, and 1-Methylecyclopropene (1-MCP) treatments on SDS development. A factorial experiment consisting of two cultivars (Williams 82, and MN1606), and seven chemical treatments: ethephon (2-chloroethyl phosphonic acid, Sigma-Aldrish, St. Louis, MO, USA) at concentrations of 0.1, 1, and 4 mM [13, 26], cobalt chloride (Fisher Scientific, Pittsburg, PA) at concentrations of 0.1, and 1 mM, and 1-MCP (Agro Fresh Inc, Philadelphia, PA) at a concentration of 24.4 mM. Ethephon is an ethylene biosynthesis inducer, cobalt chloride is an ethylene biosynthesis suppressor, and 1-MCP inhibits ethylene perception [27]. Ethephon and cobalt chloride concentrations were chosen based on preliminary results obtained from a phytotoxicity assay, while 1-MCP concentration was chosen following the company’s instructions (Agro Fresh Inc, Philadelphia, PA). The experimental units were arranged in a randomized complete block design with a total of seven blocks, with one replication per block. The experiment was conducted three times.

The chemical treatments were applied when soybean seedlings were at VC stage (first unifoliate). Ethephon and cobalt chloride were applied as a soil drench using 15 ml per pot, and 1-MCP was applied by spraying the leaves until runoff. Control plants were treated in the same way but applying a soil drench or spraying with SDW. All compounds were dissolved in SDW. Twenty-four hours after treatment application, seedlings were transplanted in *Fv* infested soil, prepared by mixing the sand-cornmeal *Fv* inoculum with pasteurized sand: soil mixture (2:1) at a ratio of (1:15) inoculum to sand-soil mixture (v/v). Twenty-four hours after transplanting, a second chemical treatment application was applied to each plant. The plants were incubated in a greenhouse bench at 24°C, with a 16-h photoperiod, watered as needed, and fertilized once a week.

#### Disease and plant growth assessments

Twenty-one days after transfer to *Fv* infested soil, plants were destructively sampled and the roots were thoroughly washed with running tap water to remove soil particles. Seven replicate cups per treatment combination were visually assessed for severity of root rot and foliar symptoms, measured as the percent of root area showing brown or black discoloration and the percent of leaf area showing chlorosis and necrosis typical to SDS, respectively. Root and shoot lengths of infected plants were measured on fresh plants, and dry weight of shoots and roots was measured after drying in at oven at 70°C for 48 h.

### Effect of chemical treatment on the growth of *Fv in vitro*

To investigate the effect of ethephon and cobalt chloride on *Fv* growth and development, full strength PDA media with antibiotics [tetrachlorocycline (0.15g/L) and streptomycin (0.15g/L)] was prepared and supplemented with ethephon (final concentrations: 1, 2, and 4 mM) or cobalt chloride (final concentrations: 0.1 and 1 mM). A 4-mm diameter plug of a twenty-eight-day-old *Fv* culture was placed, with the mycelium side facing down, in the center of petri dishes (100 mm × 15 mm) containing approximately 20 ml of amended PDA. A total of ten plates per treatment were arranged in a completely randomized design. Plates were incubated at room temperature (24°C) for 14 days under dark conditions. At the end of the incubation period, *Fv* colony diameter was measured in two perpendicular directions on each plate. Each plate was flooded with 5 ml of SDW, filtered with two layers of cheesecloth, and the conidial concentration was counted using a hemocytometer. The experiment was repeated three times.

### Expression of ethylene pathway and defense-related genes

To determine the effect of chemical treatments on soybean defense-related genes (listed in Table 1) in the presence or absence of the fungus, a factorial experiment was conducted consisting of two cultivars (Williams 82 and MN1606), and four chemical treatments: water, ethephon 4 mM, cobalt chloride 1 mM, and 1-MCP 24.4 mM. The experimental units were arranged in a randomized complete block with a total of five blocks each with two replications, and the experiment was conducted twice. Plants were chemically treated and infected with *Fv* as described above. Whole root tissue was sampled at 0 (uninfected control), 2, and 4 days post inoculation (DPI), the roots were carefully rinsed with tap water to remove soil particles, immediately frozen in liquid nitrogen, and then stored in -80°C until use. Each sample consisted of two roots pooled from each of two cups (two replication cups per block).

**Table 1:** Oligonucleotide primers used for quantitative real-time PCR.

Gene Name^a^	Sequence (5'-3')		
	Forward	Reverse	References
***Actin***	TCCAAGGGGACCTAACGGAGA	TGGGTCAAGAGCTGGATGGTG	[23]
***PR10***	GCCCAGGAACCATCAAGAAG	CGCTGTAGCTGTATCCCAAG	[26]
***ERF***	GCTTAAGGAGATGAACTATGCAAA	TAATCAGCACCAGGCATGTC	[26]
***ACO***	CATGTTTTTCGCGTTCTCCT	AAGTACAGAAAGAAAGGGATGGA	[26]
***PR1***	TGTGTTGTGTTTGTTAGGGTTAGTCA	TGTTGGTGAGTCTTGAGCATACG	[28]
***PR2***	GTCTCCTTCGGTGGTAGTG	ACCCTCCTCCTGCTTTCTC	[28]
***PR3***	GCACTTGGTCTGGATTTG	GGCTTGATGGCTTGTTTC	[28]
***CHS***	AGGCTGCAACTAAGGCAATC	TAATCAGCACCAGGCATGTC	[28]
***IPER***	CTCTCAGGTGCTCATACATTCG	TGGATCAGGTTTGCCAGTTC	[28]
***ACS***	CTTAGGCTCAGTTTCTCTTCAAGGATATTTGAT	CGCTCGAGTAGAACCCAGATCCAATC	[29]

^a^ *PR1* = PR1a precursor antimicrobial protein, *PR2* = β-1,3-Endoglucanase, *PR3* = Chitinase class I, *PR10* = Intercellular pathogenesis-related, *CHS* = Chalcone synthase, *IPER* = Basic peroxidase, *ERF* = Ethylene response factor 1, *ACO* = 1-aminocyclopropane-1-carboxylic acid oxidase, *ACS* = 1-aminocyclopropane-1-carboxylic acid synthase, *Actin* = Soybean β-actin.

For RNA extraction, root samples were ground in liquid nitrogen to a fine powder, then total RNA was extracted using RNeasy mini kit (Qiagen, Germantown, MD, USA). DNA was eliminated using RNase-free DNase I (Invitrogen, Carlsbad, CA, USA) following the manufacture’s procedure. RNA quantity was determined using a Nanodrop 1000 spectrophotometer (Thermo Scientific, Wilmington, DE, USA), and integrity was verified on 1% agarose gel. For cDNA synthesis, 0.5 μg of total RNA was reverse transcribed using SuperScript III and oligo-dT primer (Invitrogen, Carlsbad, CA, USA). The cDNA was then diluted to a final concentration of 2.5 ng/ μl. Real-time PCR was performed using the Perfecta SYBER Green fast mix (Applied Biosystems, Foster City, CA) and the iQ5 detection system (Bio-Rad, Hercules, CA, USA). The reaction mix consisted of 10 μl master mix, 0.5 μl reverse and forward primers (250 nM final concentration, Table 1), 8 μl of diluted cDNA, and the final volume was adjusted to 20 μl with RNase DNase free water (Invitrogen, Carlsbad, CA, USA). The cycling protocol consisted of 3 min at 95°C, 40 cycles of 10 s at 95°C, 15 s at primer annealing temperature (Table 1), and 30 s at 72°C. Melting curve data was collected to check for non-specific amplification and primer dimers. Each treatment had 4 or 5 biological replicates, with two technical replicates. Relative gene expression was calculated using the 2^-ΔΔ ct^ method [30] in which, the water treated control was used as the calibrator and *β-actin* as the reference gene.

### Data analyses

For the greenhouse experiment, analysis of variance was performed using the PROC GLIMMIX procedure of SAS version 9.3 (SAS institute, Cary, NC) to determine the effects of treatments on root rot severity, foliar disease severity, *Fv* inoculum density in soil, gene expression, and root and shoot dry weight and length. Chemical application and cultivar were used as a fixed effect, and block and run were used as random effects.

For the *in vitro* experiment, analysis of variance was performed using the PROC GLIMMIX procedure to determine the treatment effect of chemical on *Fv* colony diameter and number of conidia. Chemical application was used as a fixed effect, and replication and run were used as random effects. Fisher’s protected least significant difference test (*P*<0.05) was used to detect significant differences between treatments.

## Results

### *Fusarium virguliforme* infection triggers ethylene biosynthesis in soybean

**Fig 1:**
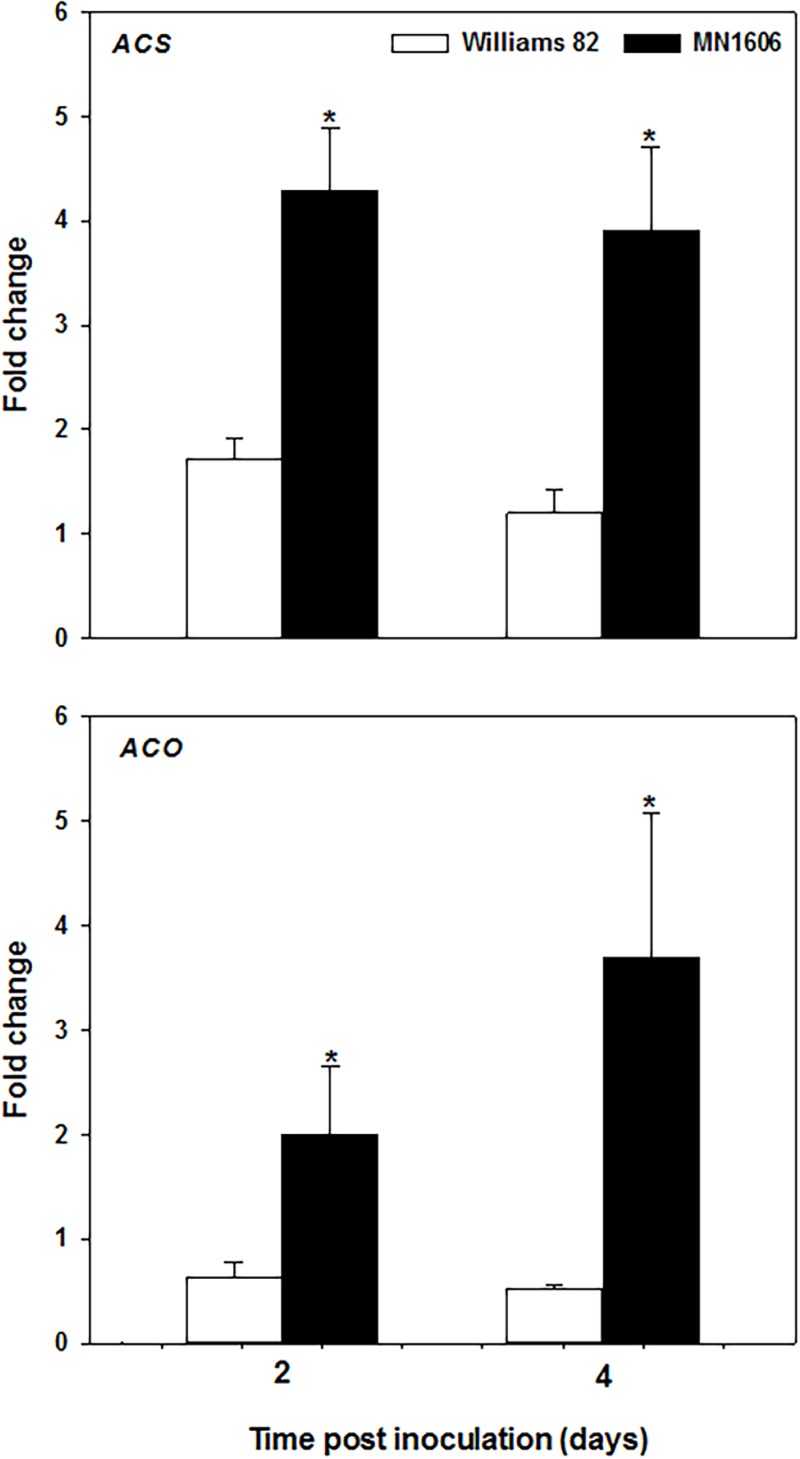
Induction of ethylene biosynthesis genes in response to *Fusarium virguliforme* infection. Expression analysis of 1-aminocyclopropane-1-carboxylic acid synthase (*ACS*), and 1-aminocyclopropane-1-carboxylic acid oxidase (*ACO*) genes in response to *Fusarium virguliforme* infection in soybean roots at 2 and 4 days post inoculation (DPI). Soybean seeds of MN1606 (resistant) and Williams 82 (moderately susceptible) were planted in *Fv* infested soil and roots were collected at 2 and 4 DPI for quantification of *ACS* and *ACO* gene expression using real time PCR. The soybean *actin* gene was used as an internal control. The level of expression is shown relative to that of time zero plants (non-inoculated). Each column represents the mean fold change of six biological samples (3 replicates × 2 runs), and each sample consisted of a pool of two plants. Error bar indicates standard error of the mean (n = 6). * indicates a significant difference (*P*<0.05) between the two cultivars within the same time point.

To examine whether *Fv* inoculation could induce ethylene biosynthesis in SDS resistance and susceptible soybean cultivars, the expression of key ethylene biosynthesis genes, 1-aminocyclopropane-1-carboxylic acid synthase (*ACS*), and 1-aminocyclopropane-1-carboxylic acid oxidase (*ACO*), were quantified in roots at 2 and 4 DPI and compared to time 0 (non-inoculated control). At both time points, the resistant cultivar MN1606 exhibited a significant (*P*<0.05) up-regulation in *ACS* and *ACO* gene expression compared to the susceptible one (Fig 1). Moreover, at early and mid-vegetative soybean growth stages (V1, and V4-V5) the expression of *ACS* gene was significantly higher in MN1606 compared to Williams 82 in response to *Fv* infection, whereas no significant difference was observed between the two cultivars at the reproductive stage (R1-R2). These results suggest that ethylene biosynthesis might be important in resistance against SDS (S1 Fig).

### Effect of chemical treatments on SDS and plant growth

#### Foliar and root rot severity

**Fig 2:**
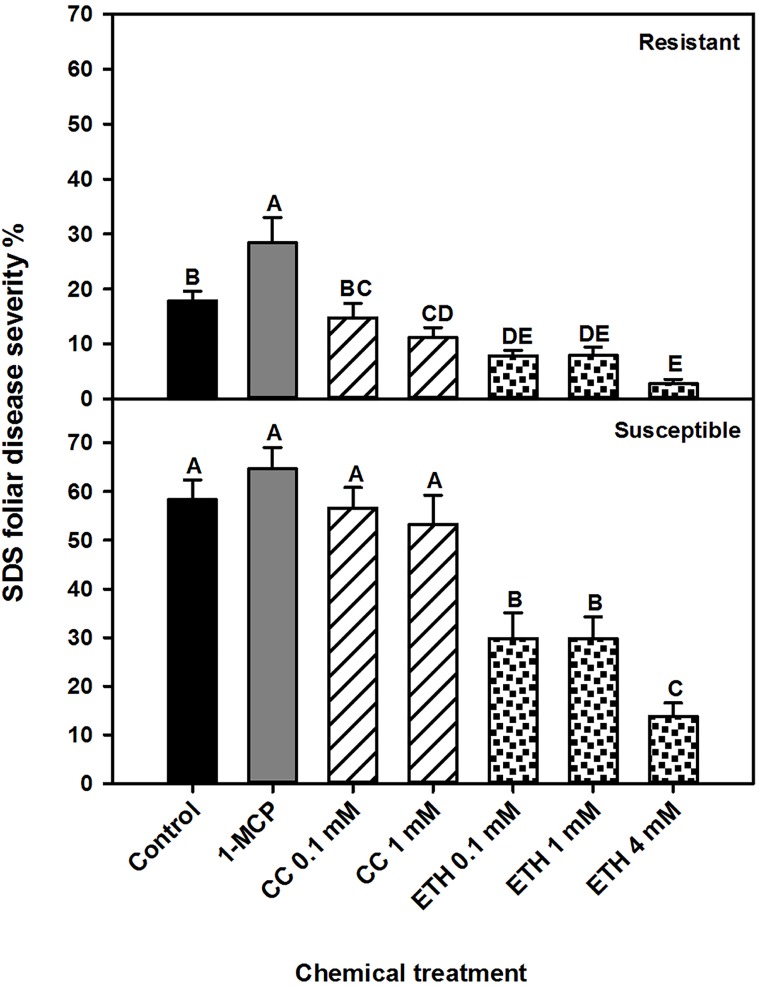
Effect of chemical treatments on severity of foliar symptoms of soybean sudden death syndrome in resistant cultivar MN106 and susceptible cultivar Williams 82. Soybean seedlings were drenched with water (control), ethephon (ethylene inducer) at concentrations of 0.1 mM, 1 mM, and 4 mM, or cobalt chloride (ethylene suppressor) at concentrations of 0.1 mM, and 1 mM, 24 h before and 24 h after transplant into soil infested with *Fusarium virguliforme*. 1-MCP (ethylene perception suppressor) was sprayed until run off at the same times. SDS symptoms were assessed 21 days post inoculation (DPI). Each bar represents the mean of 21 replicates (7 cups × 3 runs), and the error bar represents the standard error of the mean. Means with different letters are significantly different (*P*<0.05).

To investigate the role of ethylene in resistance against *Fv*, we used a pharmacological approach to either suppress or induce ethylene signaling in MN1606 and Williams 82 soybean cultivars. Chemical treatment and soybean cultivar significantly (*P*<0.001) affected SDS foliar symptoms and their interaction was significant. Therefore, the analysis is presented separately by cultivar. In both cultivars, plants drenched with ethephon at concentrations of 0.1, 1, or 4 mM exhibited a significant reduction in SDS foliar symptoms compared to the water treated control (Fig 2). In contrast, plants sprayed with 1-MCP showed significant increases in SDS foliar symptoms compared to all treatments only in cultivar MN1606, whereas no significant difference was observed in cultivar Williams 82 (Fig 2). The application of a cobalt chloride drench at 0.1 mM concentration showed no significant effect on SDS foliar symptoms compared to controls, while a small reduction was observed after 1 mM treatment in the MN1606 cultivar. There were no significant differences in root rot severity between chemical treatments and the water control (S2 Fig).

#### Root and shoot dry weight and length

In this study, we tested whether chemical treatments had an effect on the *Fv*-dependent growth reduction in soybean. There were significant chemical treatment and cultivar main effects in all growth parameters tested, and significant interactions between chemical treatment and cultivar; therefore, the analysis is presented separately by cultivar. In cultivar MN1606, *Fv*-infected seedlings treated with ethephon at 0.1 mM showed higher shoot and root dry weights compared to the water control, whereas treatments with ethephon at 1 or 4 mM showed no effect on dry weights. Cultivar Williams 82 root dry weight did not differ significantly among treatments, whereas shoot dry weight was greater in seedlings treated with ethephon at 0.1 mM compared to ethephon at 4 mM. However, 0.1 and 4 mM ethephon treatments showed no significant difference compared to the water control. Soybean seedlings treated with cobalt chloride or 1-MCP, showed no significant difference compared to control in both cultivars, except for seedlings of cultivar MN1606 treated with 0.1 mM cobalt chloride that showed higher shoot dry weight compared to 1-MCP and control seedlings (Table 2).

**Table 2:** Effect of chemical treatment on soybean growth.

	Growth parameter ^w^							
Cultivar ^y^	RDW ^x^		SDW		RL		SL	
	R	S	R	S	R	S	R	S
Treatment ^z^								
Control	0.24 b	0.22 a	0.39 bc	0.52 ab	15.19 a	13.86 ab	12.41 b	8.78 b
E0.1	0.31 a	0.22 a	0.50 a	0.57 a	13.3 ab	12.84 abc	13.36 ab	9.7 ab
E1	0.25 ab	0.22 a	0.42 abc	0.52 ab	13.9 a	11.75 c	15.10 a	9.46 ab
E4	0.22 b	0.20 a	0.36 c	0.47 b	12.8 b	12.38 bc	12.75 ab	9.7 ab
CC0.1	0.26 ab	0.22 a	0.50 a	0.50 ab	15.25 a	13.31 ab	13.87 ab	8.75 b
CC1	0.25 ab	0.24 a	0.47 ab	0.59 a	14.9 ab	13.95 a	12.87 ab	10.86 a
1-MCP	0.20 b	0.22 a	0.41 abc	0.53 ab	13.8 ab	13.90 ab	12.87 ab	9.03 b

^w^ Growth parameter: RDW = root dry weight, SDW = shoot dry weight, RL = root length, and SL = shoot length

^x^ data represents the mean of fourteen replications (seven replications × two runs). Numbers followed by different letters within the same column are significantly different at *P*<0.05 according to Fisher’s least significant difference test.

^y^ Cultivar: R = resistant cultivar MN1606, S = susceptible cultivar Williams 82

^z^ Treatment: Control = water treated, E0.1 = ethephon 0.1 mM, E1 = ethephon 1 mM, E4 = ethephon 4 mM, CC0.1 = cobalt chloride 0.1 mM, CC1 = cobalt chloride 1 mM, and 1-MCP = methylcyclopropene 24.4 mM

Ethephon treatment significantly reduced root length compared to the control when applied at a rate of 4 mM in cultivar MN1606, and at a rate of at 0.1 mM in cultivar Williams 82. Seedlings treated with ethephon at 1 mM showed greater shoot length compared to the control in cultivar MN1606, but no treatment effects were observed in Williams 82. Cobalt chloride and 1-MCP treatments did not affect root or shoot length, except in cultivar Williams 82, where seedlings treated with cobalt chloride at 1 mM showed greater shoot length compared to MCP and the control (Table 2).

### Effect of chemical treatment on *in vitro Fv* colony growth and sporulation

**Fig 3:**
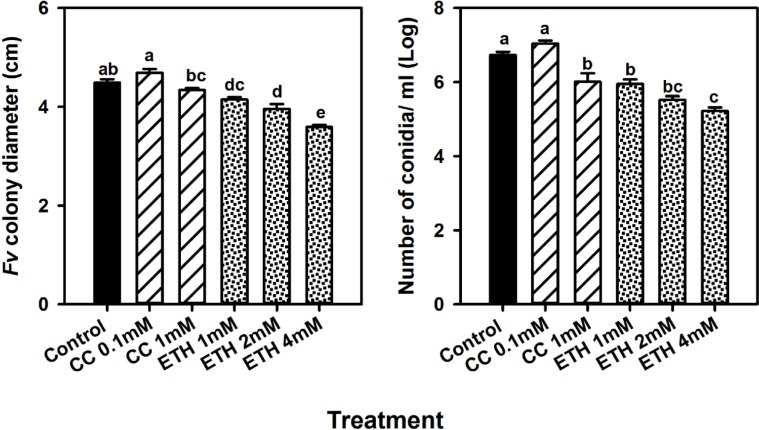
In-vitro effect of ethephon and cobalt chloride on mycelial growth *of Fusarium virguliforme*. Mycelia plugs were grown for fourteen days on PDA plates containing water, ethephon concentrations of 1mM, 2mM, and 4mM, or cobalt chloride concentrations of 0.1mM, and 1mM. Colony diameter (A), and number of conidia per ml (B) were measured fourteen days after incubation at room temperature under dark conditions. Each bar represents the mean of 18 replicates (6 plates × 3 runs) and the error bar represents the standard error of the mean. Means with different letters are significantly different (*P*<0.05).

To examine whether the chemical treatment has a direct impact on *Fv* growth, we measured *Fv* colony diameter and conidiation of fungi that were grown in media amended with ethephon, cobalt chloride, or un-amended (control). Analysis of variance showed a significant effect of chemical treatment on *Fv* colony diameter and conidiation. *Fv* colony diameter was significantly reduced in media amended with ethephon compared to control media (Fig 3A), colony morphology was also affected by ethephon; colonies on PDA supplemented with ethephon showed irregular shape and developed a purple to pinkish color that became more pronounced as the ethephon concentration increased. *Fv* colonies grown on PDA supplemented with cobalt chloride showed normal growth rate and colony morphology compared to the control (Fig 3A).

All ethephon concentrations significantly reduced the number of *Fv* conidia produced in each colony compared to the control. Colonies grown in media amended with cobalt chloride at 0.1 mM showed the same number of conidia as controls, whereas amendment with cobalt chloride at 1 mM reduced conidiation compared to the control (Fig 3B). However, no chemical treatment was able to suppress the conidial germination. To test the effect of chemicals on *Fv* density in soil, we collect soil samples used to grow Williams 82 plants, and a colony forming assay was used as quantification of fungus viability. At 21 DPI no significant chemical effect on *Fv* population was observed (S3 Fig).

### Effect of chemical treatment on the expression of soybean ethylene signaling and defense-related genes

#### Regulation of ethylene signaling by chemical treatments

**Fig 4:**
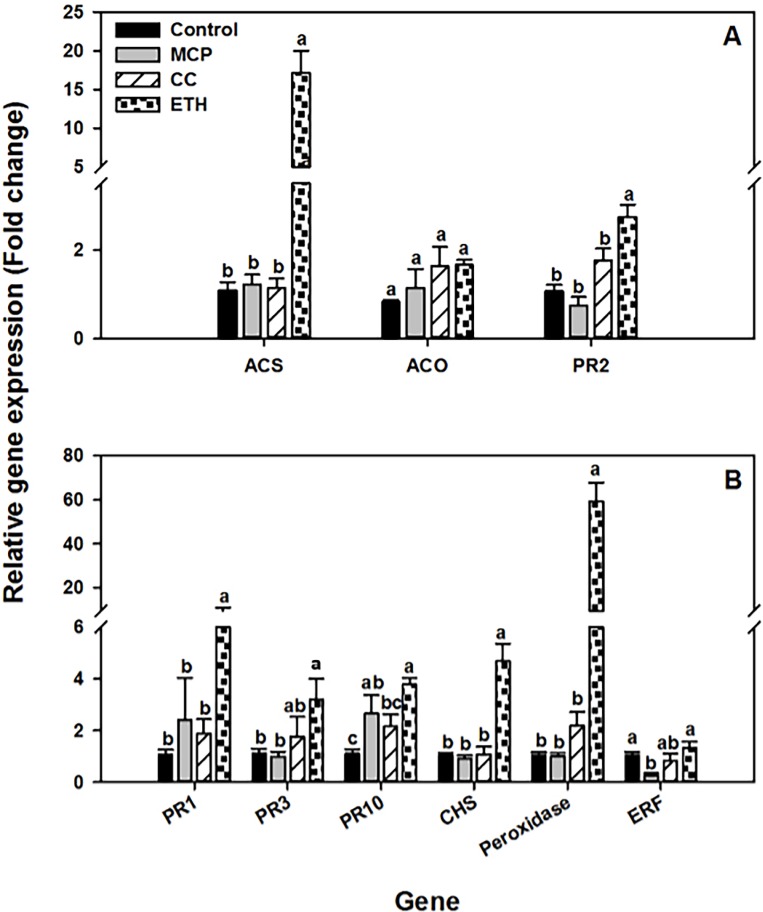
Effect of chemical treatment on defense gene expression in MN1606. Expression analysis of soybean genes twenty-four hours after exposer to water (control), 1-methylcyclopropene (MCP), cobalt chloride 1mM (CC), or ethephon 4mM (ETH) in the resistant cultivar MN1606. (A) Genes involved in ethylene biosynthesis and signaling, 1-aminocyclopropane-1-carboxylic acid synthase (*ACS*), 1-aminocyclopropane-1-carboxylic acid oxidase (*ACO*), and β-1,3-endoglucanase (*PR2*), (B) defense-related genes, pathogenesis-related protein 1 (*PR1*), Chitinase class I (*PR3*), Intercellular pathogenesis-related protein 10 (*PR10*), Chalcone synthase (*CHS*), basic peroxidase (*Peroxidase*), and ethylene response factor 1 (*ERF1*). Soybean seedlings were drenched with water (control), ethephon 4 mM, cobalt chloride 1 mM, or foliar sprayed with 1-MCP. Roots were sampled 24 hours after chemical treatment. The soybean *actin* gene was used as an internal control. The level of expression is shown relative to that of control plants. Each column represents the mean fold change of eight biological samples (four samples × two runs), and each sample consisted of a pool of two plants. Error bar indicates standard error of the mean (n = 8). Columns with different letters are significantly different (*P*<0.05).

**Fig 5:**
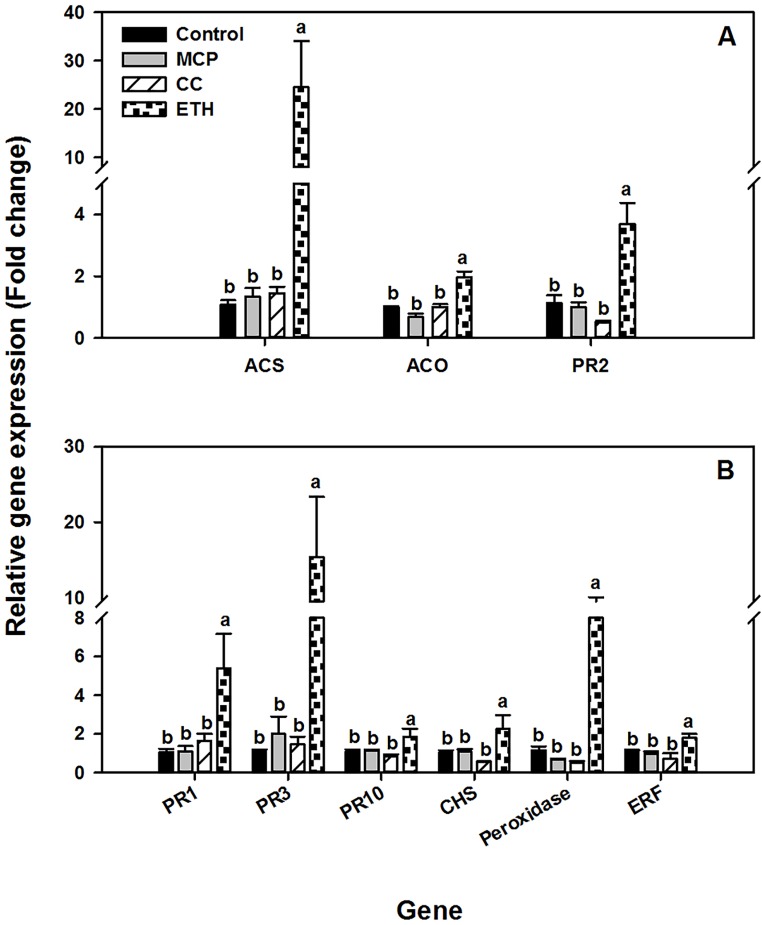
Effect of chemical treatment on defense gene expression in Williams 82. Expression analysis of soybean genes twenty-four hours after exposer to water (control), 1-methylcyclopropene (MCP), cobalt chloride 1mM (CC), or ethephon 4mM (ETH) in the moderately susceptible cultivar Williams 82. (A) Genes involved in ethylene biosynthesis and signaling, 1-aminocyclopropane-1-carboxylic acid synthase (*ACS*), 1-aminocyclopropane-1-carboxylic acid oxidase (*ACO*), and β-1,3-endoglucanase (*PR2*), (B) defense-related genes, pathogenesis-related protein 1 (*PR1*), Chitinase class I (*PR3*), Intercellular pathogenesis-related protein 10 (*PR10*), Chalcone synthase (*CHS*), basic peroxidase (*Peroxidase*), and ethylene response factor 1 (*ERF1*). Soybean seedlings were drenched with water (control), ethephon 4 mM, cobalt chloride 1 mM, or foliar sprayed with 1-MCP. Roots were sampled 24 hours after chemical treatment. The soybean actin gene was used as an internal control. The level of expression is shown relative to that of control plants. Each column represents the mean fold change of eight biological samples (four samples × two runs), and each sample consisted of a pool of two plants. Error bar indicates standard error of the mean (n = 8). Columns with different letters are significantly different (*P*<0.05).

There was a significant main effect of chemical treatment at all time points tested, and a significant interaction between chemical treatment and cultivar; therefore analysis is presented separately by cultivar. Twenty-four hours after chemical treatment the expression of the ethylene signaling markers *ACS and PR2* genes was significantly higher in roots of both soybean cultivars exposed to ethephon 4 mM application compared to the other treatments, and expression of the *ACO* gene was also elevated by this treatment in Williams 82. In contrast, 1-MCP and cobalt chloride 1mM application has no significant effect on gene expression compared to control (Figs 4A and 5A). These results indicate that soybean roots respond to ethephon treatment by enhancing the expression of ethylene responsive genes in the absence of *Fv* infection as expected.

### Effect of ethephon treatment on soybean defense genes

In both cultivars, the expression of all genes tested was significantly higher in soybean roots 24 h after treatment with ethephon 4mM compared to control, whereas cobalt chloride 1mM and 1-MCP treatments had minor effect on the expression of defense-related genes tested (Figs 4B and 5B). For instance, in cultivar MN1606, the expression level of *PR* genes were at least 3-fold higher in ethephon treated seedlings compared to control. Moreover, *peroxidase* and *CHS* gene expression were 5 and 58 fold higher, respectively, compared to control, whereas no significant difference among treatments was observed in *ERF1* gene expression (Fig 4B). In cultivar Williams 82, the same pattern was observed with few exceptions. For instance, *PR1*, *PR2*, *PR3* and *peroxidase* gene expression were higher by approximately 5, 4, 15 and 8 folds respectively compared to control, whereas *PR10*, *CHS*, and *ERF1* showed slight up-regulation compared to control (Fig 5B).

### Effect of ethephon treatment on soybean defense-related genes in response to *Fv* infection

**Fig 6:**
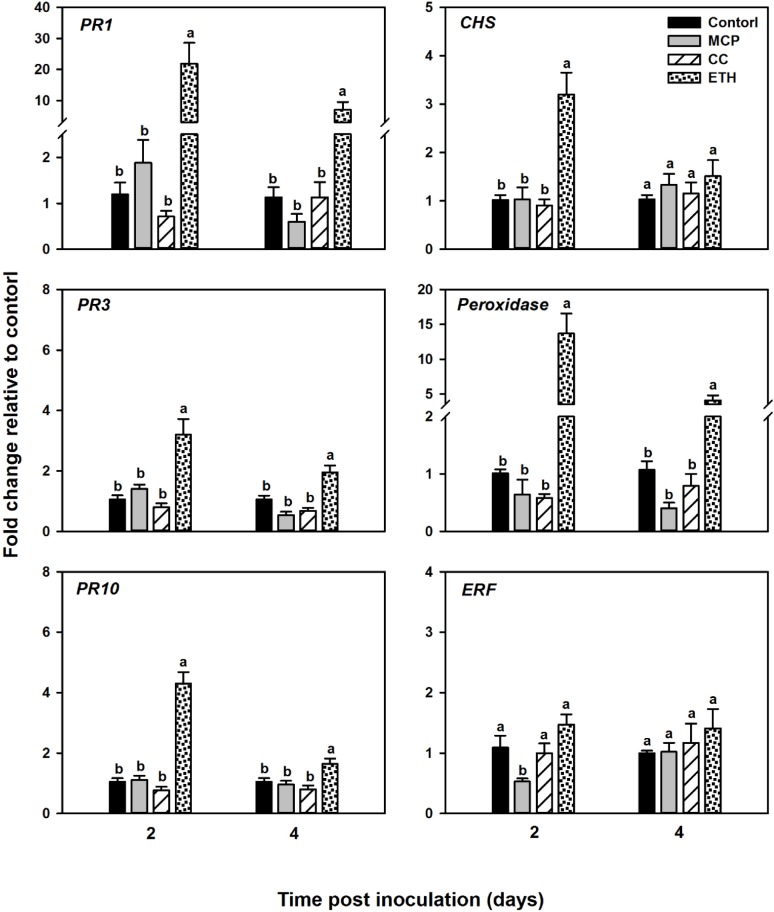
Effect of chemical treatments on the expression of soybean defense-related genes in the resistant cultivar MN1606 infected with *Fusarium virguliforme*. Soybean seedlings were drenched with water (control), ethephon 4 mM, cobalt chloride 1 mM, or foliar sprayed with 1-MCP twenty-four hours pre and post *Fusarium virguliforme* inoculation. Roots were sampled at 2 and 4 days after *F*. *virguliforme* (DAI). The level of expression of pathogenesis-related protein 1 (*PR1*), Chitinase class I (*PR3*), Intercellular pathogenesis-related protein 10 (*PR10*), Chalcone synthase (*CHS*), basic peroxidase (*Peroxidase*), and ethylene response factor 1 (*ERF1*), is shown relative to that of control plants. Each column represents the mean fold change of eight biological samples (four samples × two runs), and each sample consisted of a pool of two plants. Error bar indicates standard error of the mean (n = 8). Columns with different letters are significantly different (*P*<0.05).

**Fig 7:**
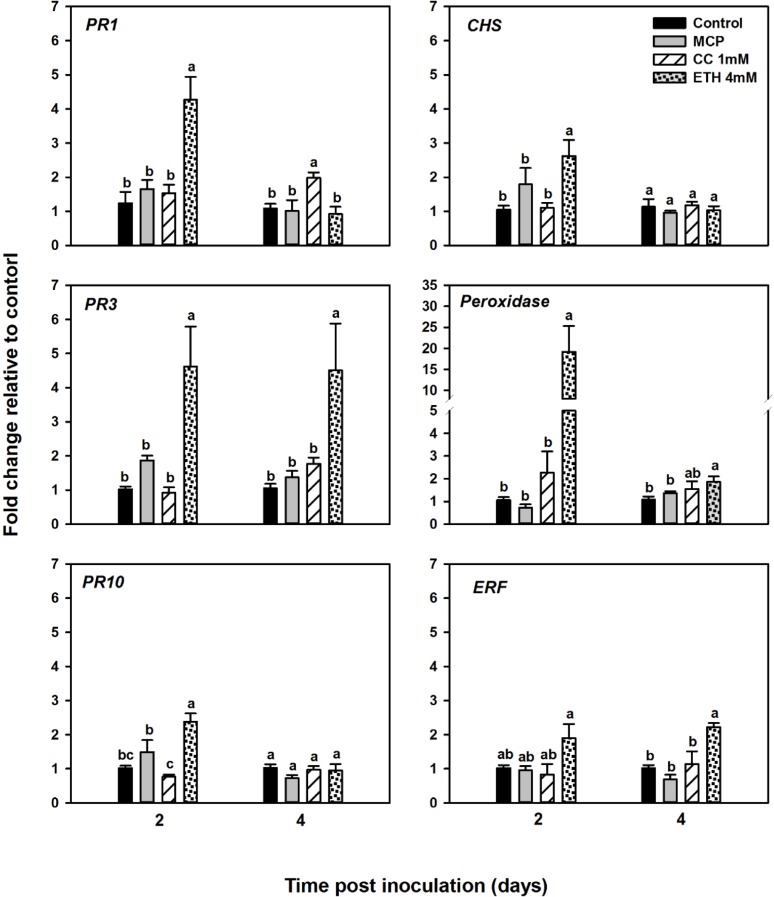
Effect of chemical treatments on the expression of soybean defense-related genes in the susceptible cultivar Williams 82 infected with *Fusarium virguliforme*. Soybean seedlings were drenched with water (control), ethephon 4 mM, cobalt chloride 1 mM, or foliar sprayed with 1-MCP twenty-four hours pre and post *Fusarium virguliforme* inoculation. Roots were sampled at 2 and 4 days after *F*. *virguliforme* (DAI). The level of expression of pathogenesis-related protein 1 (*PR1*), Chitinase class I (*PR3*), Intercellular pathogenesis-related protein 10 (*PR10*), Chalcone synthase (*CHS*), basic peroxidase (*Peroxidase*), and ethylene response factor 1 (*ERF1*), is shown relative to that of control plants. Each column represents the mean fold change of eight biological samples (four samples × two runs), and each sample consisted of a pool of two plants. Error bar indicates standard error of the mean (n = 8). Columns with different letters are significantly different (*P*<0.05).

There was a significant main effect of chemical treatment at the time points tested (2 and 4 DPI), and a significant interaction between chemical treatment and cultivar; therefore analysis is presented separately by cultivar. In both cultivars, at 2 and 4 DPI, genes encoding pathogenesis-related proteins and peroxidase gene were highly expressed in roots treated with ethephon 4mM compared to the other treatments, except at 4 DPI in Williams 82 cultivar where no significant difference was observed among treatments in *PR10* gene expression. In contrast, cobalt chloride 1mM and 1-MCP treatments showed no effect on the expression of these genes compared to control, in either cultivar at both time points (Figs 6 and 7). Moreover, the expression level of *CHS* was the highest in ethephon-treated seedlings compared to control at 2 DPI in both cultivars, however this level returned back to the control levels at 4 DPI. We also investigated the effect of chemical treatments on the expression of the transcription factors *ERF1*. Interestingly, we found that the expression of *ERF1* gene increased only in the susceptible cultivar Williams 82 after ethephon application at both time points, whereas no change was observed among treatments in the resistant cultivar MN1606 (Figs 6 and 7).

## Discussion

In this work, we investigated the effect of ethylene suppression and induction on soybean resistance against *Fv* infection. Previous studies have shown the importance of phytohormone signaling in resistance against plant diseases [31, 32], but to our knowledge, this is the first report directly testing the role of the ethylene hormone in the soybean-*Fv* interaction.

Our results showed that, *Fv* infection can triggers ethylene accumulation in resistant cultivar MN1606 at a higher level compared to the susceptible cultivar Williams 82, as indicated by the expression of ethylene biosynthesis marker genes *ACS* and *ACO*.

We also showed that induction of ethylene signaling in soybean roots using a soil drench with ethephon reduced SDS foliar symptoms development by up to 75% compared to a drench with water. In contrast, suppression of ethylene biosynthesis or perception, by application of cobalt chloride or 1-MCP, respectively, in general resulted in the same or higher SDS development compared to the control, respectively. Furthermore, a direct inhibitory effect of mycelium growth and sporulation was observed on *Fv* plugs grown on PDA media supplemented with ethephon, whereas media amendment with cobalt chloride either had no effect or reduced *Fv* growth or sporulation compared to controls. However, no chemical effect on *Fv* population density was observed in soil.

At the molecular level, drench applications of ethephon at 4mM enhanced the expression of genes involved in soybean ethylene biosynthesis and defense responses. Taken together, these results suggest that ethephon soil application is a promising inducer of ethylene signaling pathway that may play a positive role in soybean resistance against *Fv* infection.

Our findings showing reduction of SDS severity in response to exogenous application of ethephon and induction of ethylene signaling are in agreement with previous studies in other pathosystems [27]. For example, pretreatment of soybean seedlings with ACC (ethylene precursor) enhanced plant survival rate against *Phytophthora sojae* infection [26]. Similarly, ethephon treatment triggered protection of grapevine leaves against *Erysiphe necator*, the causal agent of powdery mildew [15], and transgenic rice with inducible ethylene production was more resistant to *Magnaporthe oryzae* and *Rhizoctonia solani* [33]. Furthermore, ethylene insensitive soybean lines were more susceptible to white mold caused by *Sclerotinia sclerotiorum* compared to wild type in field conditions [19].

In our study, *Fv* cultures grown on PDA media supplemented with ethephon showed reduced colony size, reduced sporulation and different morphology compared to cultures grown in non-amended media. This result is consistent with a report of delayed growth of *Phytophthora capsici*, the causal agent of Phytophthora blight in bell pepper, on PDA media supplemented with 5mM ethephon [13]. However, in contrast to the inhibitory effect observed *in-vitro*, ethephon had no effect on *Fv* population in soil (S3 Fig).

It has been well documented that plants defend themselves against pathogen attack by inducing defense responses that are modulated by phytohormones [31]. However these hormone-regulated defenses are often accompanied by fitness costs [34, 35]. For example, the Arabidopsis mutant *cev1* that constitutively expresses the jasmonic acid and ethylene pathways is resistant against powdery mildew, but also shows a stunted phenotype [36]. In our study, application of ethephon affected root and shoot dry weight and length, however, this effect was dependent on cultivar and dose. For example, in cultivar MN1606, the greatest shoot and root dry weight were observed at the lowest ethephon concentration, whereas no effect was observed among the other concentrations or in cultivar Williams 82. Also, root length was decreased by some ethephon treatments in both cultivars. This is consistent with a study done by Urwiler and Stutte who found that application of ethephon at high rate affected soybean seed and pod development, whereas low rate application had no effect [37].

Furthermore, in our study we found that low cobalt chloride at concentrations of 0.1 or 1 mM had little effect on soybean growth compared to the control, whereas a phytotoxic effect was observed on leaves treated with high concentration of cobalt chloride 10 mM. These results were consistent with earlier studies [38–40] showing that lower cobalt (50mg/kg) can increase soybean growth and nodulation, while high doses adversely affect these parameters. It has been reported that results from cobalt treatments to inhibit ethylene production should be taken carefully because Co also has ethylene-independent toxic effects on plants [41]

At the molecular level, it is known that manipulation of the ethylene pathway using pharmacological or genetic approaches induces the expression of plant defense-related genes and enhances resistance against different biotic and abiotic stresses [27, 42]. For instance, exogenous application of ethephon enhanced resistance to charcoal rot in *Medicago truncatula* [17], *Phytophthora sojae* in soybean [26], *Phytophthora capsici* in Habanero pepper [13], and *Erysiphe necator* in grapevine [15]. This increased resistance was probably due to accumulation of pathogenesis-related proteins and antimicrobial compounds such as phytoalexins [14]. Furthermore, ethylene insensitivity in tobacco impaired resistance against soilborne fungi [16] and an ethylene insensitive pea mutant *ein2* developed more severe symptoms when challenged by *Fusarium oxysporum* or *Pythum irregulare* compared to wild type peas [43, 44]. Similarly, our study showed that induction of ethylene signaling in soybean roots using ethephon enhanced resistance against SDS foliar symptoms and increased accumulation of key defense response genes such as *CHS*, and basic peroxidase *IPER*, compared to water treated control.

Ethephon application also induced the expression of various pathogenesis related-proteins (PR) genes such as *PR1*, *PR2*, *PR3*, and *PR10* that encode enzymes with antifungal activity, as previously reported [45, 46]. The *PR1* gene is commonly used as a marker for salicylic acid signaling and systemic acquired resistance [47]. In agreement with our results, Nunez-Pastrana et al [13] showed a direct correlation between the survival of ethephon-treated Habanero pepper seedlings against *Phytophthora capsici* and the accumulation of the *PR1* gene. The *PR2* and PR3 genes code for a β-1,3 endoglucanase and a class I chitinase, respectively; these enzymes contribute to plant defenses by hydrolyzing fungal cell wall components such as β-1,3-glucans and chitin, respectively [48, 49]. Transgenic Indian cotton expressing a rice chitinase gene displayed resistance to *F*. *oxysporum* and *Alternaria macrospora* infection [50]. Furthermore, ethylene pretreatment increased *PR2* and *PR3* activity and induced resistance against *Botrytis cinerea* in tomato and *Phytophthora megasperma* in soybean [51, 52].

Another important host defense mechanism involves the rapid accumulation of reactive oxygen species (ROS) in response to pathogen attack, a phenomenon called oxidative burst [53]. This reaction is directly toxic to pathogens and can lead to a hypersensitive response that prevents further pathogen spread. In our study, we did not measure ROS levels but we quantified the expression of the peroxidase gene *IPER*. Peroxidases are involved in ROS regulation, cell wall lignification, and in defense response against pathogen attack [54]. Our data showed a strong induction of *IPER* 24 hours after ethephon application compared to the water-treated control. This result is in agreement with Yi and Hwang who demonstrated that *IPER* accumulated in soybean roots in response to ethephon treatment, and that *IPER* accumulation was greater in soybean hypocotyls infected with an incompatible race of *P*. *sojae* compared to low levels in the compatible interaction [55].

In contrast to the positive role of ethylene induction on resistance against SDS, suppression of ethylene biosynthesis by cobalt chloride had no effect on SDS foliar development. However, in one of the two cultivars tested, blocking of ethylene perception by 1-MCP resulted in more severe SDS symptoms compared to water or ethephon-treated seedlings, supporting a possible role of ethylene perception in resistance against *Fv* infection. Similarly in tomato, silencing of multiple *ACS* genes that are involved in ethylene biosynthesis did not affect *Mi-1*-mediated resistance against root-knot nematode infection. However, the tomato Never ripe (*Nr*) mutant that is compromised in ethylene perception was more attractive to the infective juveniles and showed enhanced susceptibility to RKN infection [56]. These results suggest that redundancies in the ethylene biosynthetic pathway exist, or reduced levels of ethylene are still sufficient to trigger a response, while blocking the signaling pathway may be more effective as a tool to identify the role of ethylene in different plant responses, and it would be a good approach to follow up the analysis of ethylene’s role on the soybean-*Fv* interaction.

## Conclusions

The results of this study support our hypothesis that the ethylene-signaling pathway is important in resistance against SDS. We observed a correlation between ethylene signaling, accumulation of defense-related genes, and SDS resistance in response to ethephon treatment. A limitation of our study is that ethephon was applied to soybean seedlings at VC stage, before *Fv* infection, and under controlled environmental conditions. Future work should validate the effect of ethephon on SDS under field conditions and at different application times. If ethylene is shown to enhance resistance against *Fv* infection in field conditions, then transcriptomic analysis of soybean seedlings in response to ethephon treatment is needed to identify resistant genes that could be incorporated into breeding against *Fv*.

## Supporting information

S1 Fig Expression analysis of 1-aminocyclopropane-1-carboxylic acid synthase (*ACS*) gene in response to *Fusarium virguliforme* infection at different soybean growth stages. Soybean seeds of MN1606 (resistant) and Williams 82 (susceptible) were planted in *Fv* infested soil and roots were collected at V1, V4-V5, and R1-R2 soybean growth stages for quantification of *ACS* gene expression using real time PCR. The soybean actin gene was used as an internal control. The level of expression is shown relative to that of control plants (non-inoculated) at each corresponding soybean growth stage. Each column represents the mean fold change of three biological samples, each sample was a pool of two plants. Error bar indicates standard error of the mean (n = 3). * indicates a significant difference (*P*<0.05) between the two cultivars within the same growth stage.
(EPS)

S2 Fig Effect of chemical treatments on severity of root rot symptoms in soybean cultivars. Soybean seedlings were drenched with water (control), ethephon (ethylene inducer) at concentrations of 0.1 mM, 1 mM, and 4 mM, or cobalt chloride (ethylene suppressor) at concentrations of 0.1 mM, and 1 mM, 24 h before and 24 h after transplant into soil infested with *Fusarium virguliforme*. 1-MCP (ethylene perception suppressor) was sprayed until run off at the same times. Root rot symptoms were assessed 21 days post inoculation (DPI). Each column represents the mean of 21 replicates (7 cups x 3 runs), and the error bar represents the standard error of the mean. Means with same letters are not significantly different (*P*>0.05).
(EPS)

S3 Fig Population density of *Fusarium virguliforme* in soil. Soybean seedlings (Williams 82) were drenched with water (control), ethephon (ethylene inducer) at concentrations of 0.1 mM, 1 mM, 2 mM and 4 mM, or cobalt chloride (ethylene suppressor) at concentrations of 0.1 mM, and 1 mM, 24 h before and 24 h after transplant into soil infested with *Fusarium virguliforme*. Soil samples were collected 21 days post inoculation (DPI) and colony forming analysis was performed. Each bar represents the mean of 4 replications, and the error bar represents the standard error of the mean. Means with same letters are not significantly different (*P*>0.05).
(EPS)

S1 Dataset Raw data used for the analysis and calculations of treatment means shown in each of the figures and tables in the manuscript. Data for each figure and table is shown in separate worksheets within the excel file.
(XLSX)
